# Supplementary material for: Innovative Fabrication of Hollow Microneedle Arrays Enabling Blood Sampling with a Self-Powered Microfluidic Patch
Source: Micromachines (Basel). 2023 Mar 7;14(3):615. doi: 10.3390/mi14030615 (PMC10052199; doi:10.3390/mi14030615)
Supplement: Supplementary file 1 [file micromachines-14-00615-s001.zip › micromachines-2204591-supplementary.pdf]

Supplementary information

Innovative Fabrication of Hollow Microneedle Arrays Enabling Blood Sampling with a Self-Powered Microfluidic Patch

Lorenz Van Hileghem [1,2], Shashwat Kushwaha [2,3,4], Agnese Piovesan [5], Pieter Verboven [5], Bart Nicolaï [5], Dominiek Reynaerts [2,3,4], Francesco Dal Dosso [1,2] and Jeroen Lammertyn [1,2,*]

[1] Biosensors Group, Department of Biosystems, KU Leuven, Willem de Croylaan 42, 3001 Leuven, Belgium
[2] Institute of Micro- and Nanoscale Integration, KU Leuven, 3001 Leuven, Belgium
[3] Manufacturing Processes and Systems, Department of Mechanical Engineering, KU Leuven, Celestijnenlaan 300, 3001 Leuven, Belgium
[4] Member of Flanders Make, 3000 Leuven, Belgium
[5] Postharvest Group, Department of Biosystems, KU Leuven, Willem de Croylaan 42, 3001 Leuven, Belgium
* Correspondence: jeroen.lammertyn@kuleuven.be; Tel.: +32-16-32-14-59

S1 Micro-milling tool list

Table S1: List of cutting tools, obtained from DIXI Polytool (Switzerland), used for machining the bevel angle and the needle bodies.

Operation	Tool type	Cutting edges	Angle (°)	Tool material	d (mm)
**Milling**	End mill	2		Tungsten carbide	0.40
**Milling**	End mill	2		Tungsten carbide	1.00
**Milling**	End mill	2		Tungsten carbide	4.00
**Engraving**	½ engraving tool		50	Tungsten carbide	0.15

S2 Flow characterization of the HMNAs

For the flow characterizations, a chip (design shown in Figure S1a) with a single microfluidic channel (1.5 mm wide was prepared. A circular shaped PSA piece with a 2 mm hole in the center was used to connect the chip inlet with the pressure pump via an in-house modified connector (Figure S1b-c). At the outlet, the HMNA was attached also using a PSA piece with a hole in the center smaller than the inter-needle distance. This way, the HMNA was laminated on the plastic

chip with a properly sealed connection to flow the water only through a single lumen at the time. After attaching the HMNA, the water container was filled with distilled water (Figure S1b-c). When a steady flow and resulting pressure was reached, data logging was started. For each lumen, the same was repeated without HMNA attached to obtain a baseline per measurement. Hereto, the HMNA was removed while keeping the PSA connector piece attached to the chip outlet. This way, the obtained pressures were corrected for influences of the flow through the PSA connector, microfluidic channel and tubing or the effects at the water-air interface during droplet formation at the microneedle tip.

The measurement setup (LineUp push/pull pressure controller and medium flow sensor, Fluigent, France) and how the system was connected are shown in Figure S1c. The pressure controller was connected to a falcon tube containing water, the flow sensor and the chip with HMNA attached. By the feedback loop from the flow sensor to the pressure controller, a flow of 50 µL/min was set and kept steady while logging the pressures required to maintain this flow using the Fluigent A-I-O software.

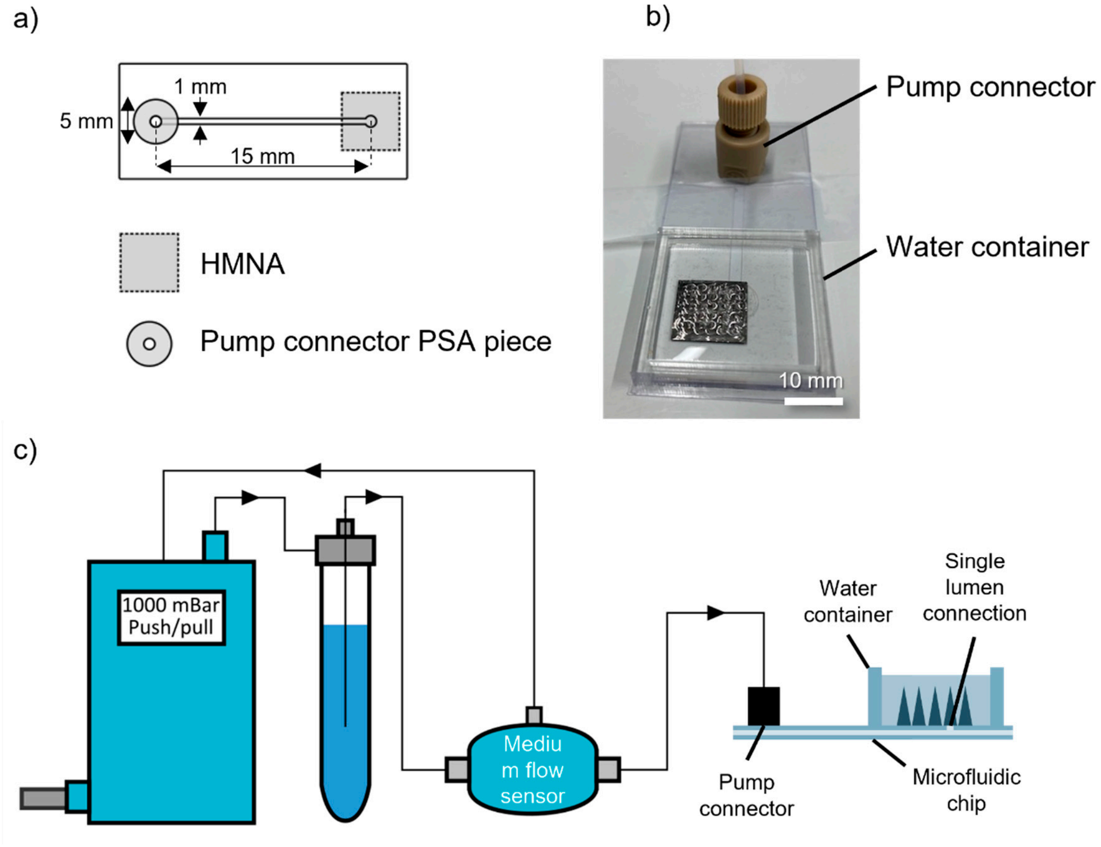

Figure S1: a) Chip design of a single microfluidic channel of 1.5 mm wide with in- and outlets of 2 mm. At the inlet, the pressure pump connector was attached by lamination with a PSA piece. At the outlet, the same concept was used for attaching the HMNA. **b)** Image of the microfluidic chip with attached HMNA and filled water container, ready for

a measurement. **c)** Schematic representation of the measurement setup used for the flow characterizations of the HMNAs.

S3 SIMPLE pressure generation measurement

An exploded view showing all the elements of the chip used for this experiment is shown in Figure S2a. This setup allowed measuring the generated pressure by the SIMPLE by expanding the volume of an air plug. The top view of a chip during this process is shown in Figure S2b. For the measurement, based on Boyle's law, it was key to maintain the air pressure in the air chamber at 101.33 kPa (atmospheric air pressure). This was done by first connecting the syringe pump (Harvard PHD 2000 syringe pump, Harvard Apparatus, USA) to the chip using a connector piece and a PSA ring. Then, the activation liquid (1/50 diluted green aqueous dye in distilled water) was prefilled until an indication to keep the same distance from the HSV for all measurements. Next, the working liquid (1/50 diluted blue aqueous dye in distilled water) was prefilled via the prefilling inlet, again until an indication mark. During working liquid prefilling, the hydrophobic barrier (HB-1) directed the working liquid towards the paper pump without compressing the air plug. Last, the working liquid prefilling inlet was sealed airtight by a piece of PSA.

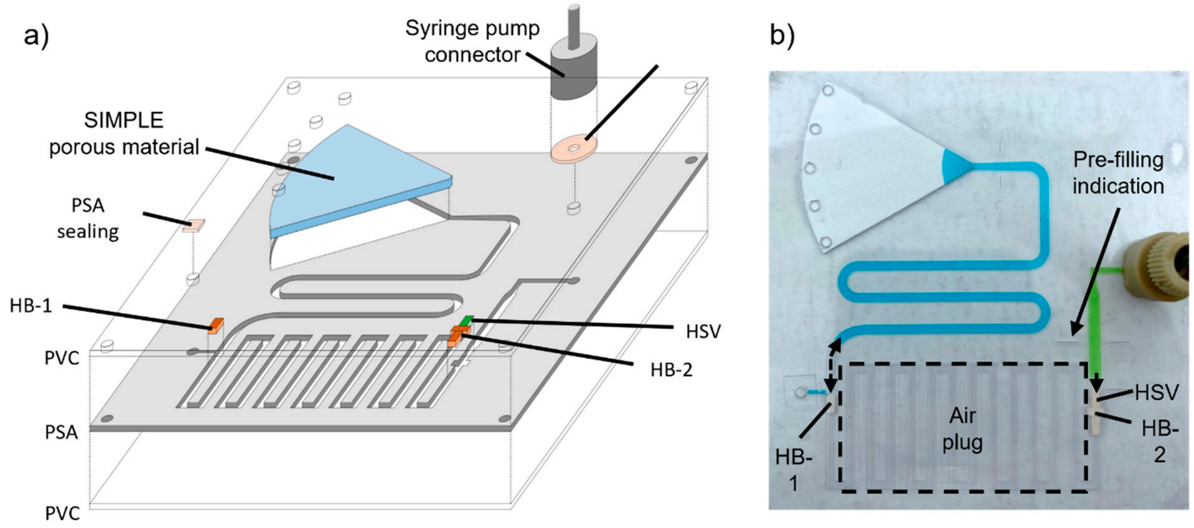

Figure S2: Chip design and top view of a chip that is being activated for SIMPLE pressure generation measurements.

Figure S3 shows (1) the measured volumes of the working liquid in the working liquid chamber, (2) the wicked in working liquid into the SIMPLE paper pump and (3) the wicking in activation liquid into the HSV. It must be noted that the second and third are just indicative as it is not possible to measure the exact volumes wicked into filter paper by video analysis. Nevertheless, these measurements are valuable as the moment on which the activation liquid saturates the HSV can be exactly measured. This was done for normalizing the measured pressures by subtracting the working liquid volume change caused by the activation instead of the action of the SIMPLE

paper pump itself. The moment at which the working liquid started moving during syringe pump activation is indicated with a blue dashed line.

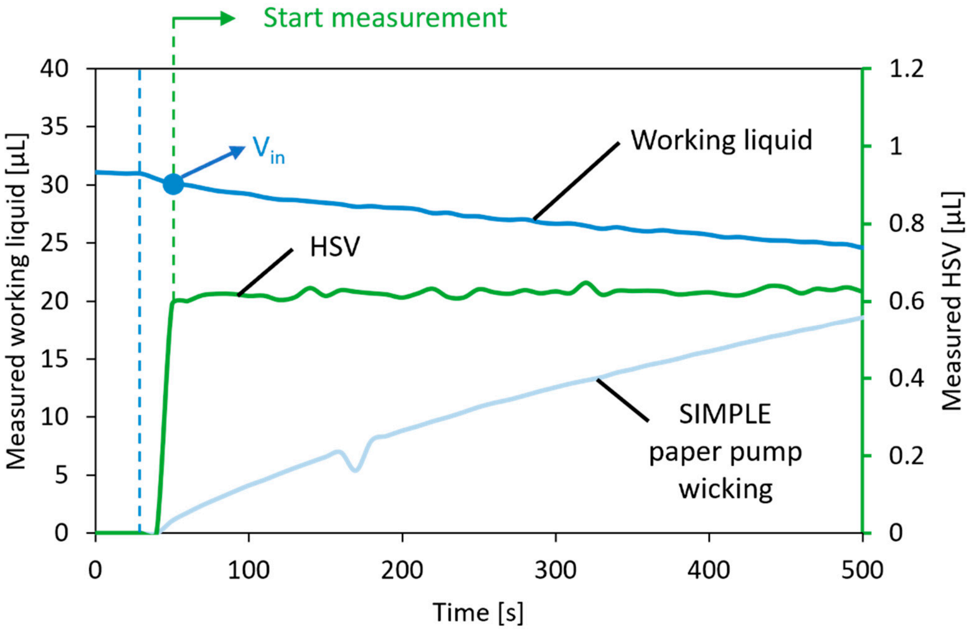

Figure S3: Measured volume of working liquid (dark blue), indicative volume of working liquid inside of the SIMPLE paper pump (light blue) and indicative volume of activation liquid into the HSV (green). The blue dashed line indicates the moment at which the working liquid starts moving toward the SIMPLE paper pump tip. The green dashed line indicates the moment at which the HSV is completely saturated, and thus from where the measurement starts. Note: the dip in the graph for the SIMPLE paper pump was caused by a video processing artefact.

The generated pressure P_{fin} [Pa], as given in Section 2.6 of the main manuscript, is calculated by filling in the working liquid volume (V_{in}) at the start of the measurement (i.e. saturated HSV) and the value of the working liquid volume during the measurement ($V(t)_{fin}$):

$$P_{fin} = \frac{P_{atm} \cdot \mathbf{V_{in}}}{\mathbf{V(t)_{fin}}} - P_{atm}$$

S4 Laser drilling settings characterization

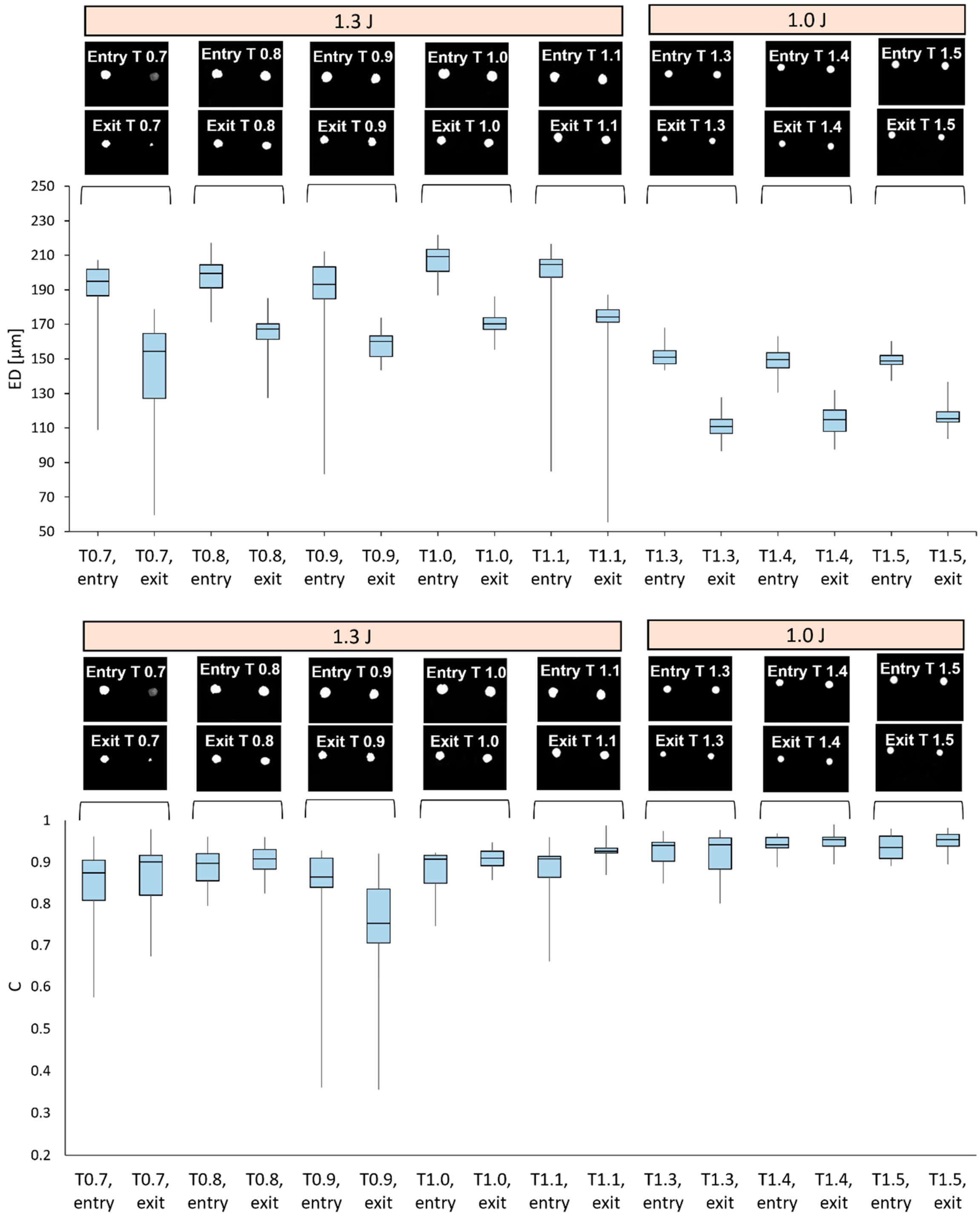

Figure S4: Complete overview of the laser drilling results on the equivalent diameter (ED) and circularity for the laser output energies 1.3 J and 1.0 J and pulse widths from 0.7 to 1.5 s. Per laser setting, an example of two obtained BF images at the laser entry and exit side are also shown. The results clearly show the improved variability when a lower laser power is used, both for the ED and the circularity results.

S5 Flow characterization of the fabricated HMNAs

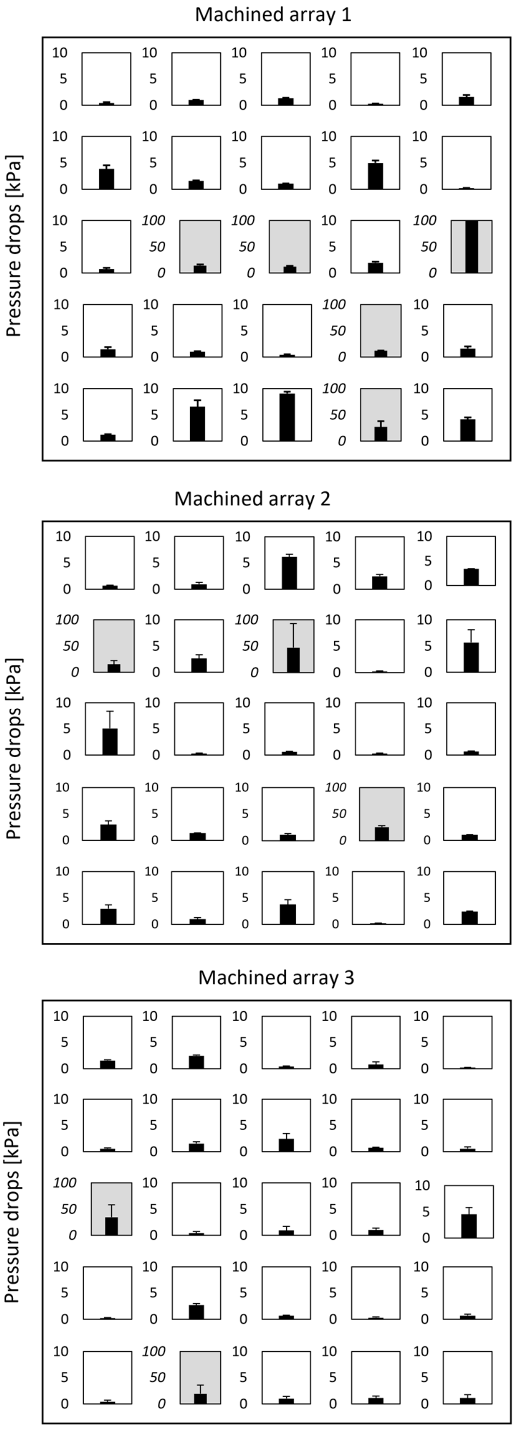

Figure S5: Bar charts of the pressure drops for distilled water flow (50 µL/min) through each individual lumen of the 3 tested HMNAs, which are represented as a heat map in the main manuscript. Bar charts with a grey background show that the y-axis (pressure drops) are shown in the range of 0-100 kPa, while the range for a white background is only 0-10 kPa. Error bars represent a single standard deviation for three repetitions.

Table S2: Results of the non-parametric Kruskal-Wallis test to show that the 3 characterized HMNAs are not significantly different from each other in terms of pressure drop. It must be noted that the results of 1 lumen on Array

1 was omitted from the data as the measured pressure exceeded the 100 kPa measurement limit for the pressure controller, resulting in a flow for which the real pressure drop could not be determined.

**Kruskal-Wallis Tests (Rank Sums)**					
**Level**	**N**	**Score Sum**	**Expected Score**	**Score Mean**	**(Mean-Mean0)/Std0**
Array 1	24	1020.00	900.00	42.50	1.38
Array 2	25	1005.00	937.50	40.50	0.77
Array 3	25	750.00	937.50	30.00	-2.14
**1-Way Test (X^2 approximation)**					
**X^2**	**DF**	**P>X^2**			
4.73	2	0.0939			

Table S3: Results of the non-parametric Kruskal-Wallis test with outliers omitted from the data (based on the quartile technique) to assure whether the conclusion is still the same.

**Kruskal-Wallis Tests (Rank Sums)**					
**Level**	**N**	**Score Sum**	**Expected Score**	**Score Mean**	**(Mean-Mean0)/Std0**
Array 1	19	682.00	617.50	35.89	0.94
Array 2	22	791.00	715.00	35.95	1.07
Array 3	23	607.00	747.50	26.39	-1.96
**1-Way Test (X^2 approximation)**					
**X^2**	**DF**	**P>X^2**			
3.86	2	0.1448			

S6 Machining artefacts

In Figure S6, extra images of the finished HMNAs are shown. Stereo-microscopy (Figure S6c) showed evidence of the creation of machining burrs (a thin material chip bending toward the inside of the lumen) that potentially restrict the microneedle lumen at the tip. However, μCT scanning did not demonstrate this and water could always pass through the lumen at high flow rates (50 μL/min).

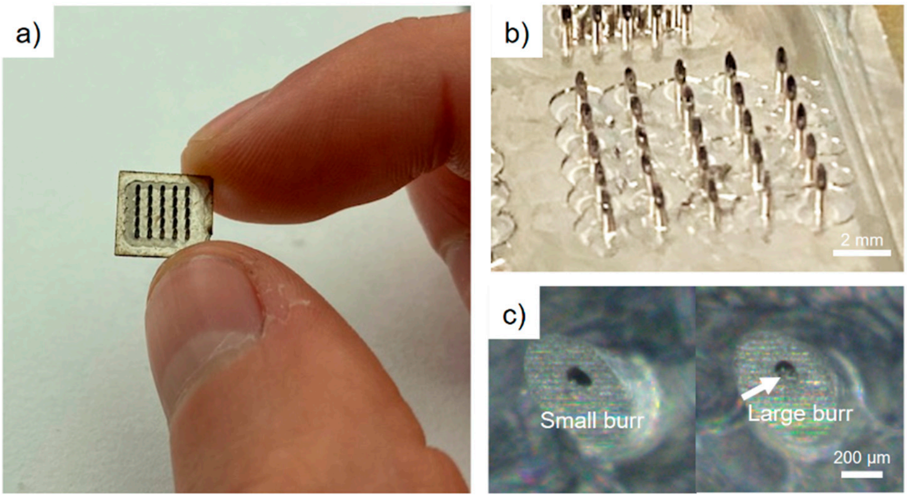

Figure S6: 5x5 HMNA a) after and b) before retrieval from the machining workpiece. c) Top view of a stereo-microscopy image showing that a lumen at the tip side seemed to be partially blocked, as indicated by the white arrow.

S7 Optimization of SIMPLE fabrication

The fabrication of the SIMPLE paper pump was optimized to overcome high pressure gradients. First, the standard fabrication protocol was explored, but pressure gradients of only 5.3 kPa were reached. Beyond this pressure, the connection between the working liquid and the porous paper tip broke. Such an event was captured by BF microscopy (see inset of Figure S7a), showing that, after a certain negative pressure gradient is achieved, air flows back via the interface between the porous paper and its chamber walls, breaking the working liquid-paper connection, and thus stopping the pump. It was expected that the lack of a tight sealing between the two material types allows this air backflow. Therefore, transfer tape was laminated on both sides of the porous paper tip to seal the filter paper pump (see inset of Figure S7b). This led to an almost 10-fold improvement of the achieved pressures.

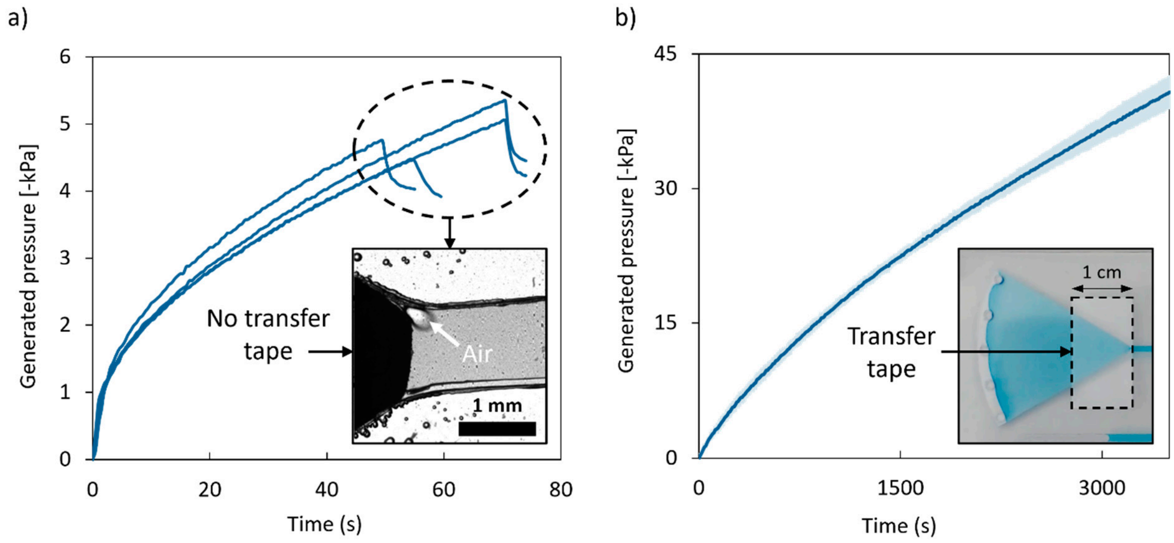

Figure S7: a) Generated pressures by the SIMPLE as fabricated without transfer tape sealing. The BF microscopy image captures the moment of pump rupture due to air leakage along the interface between the porous paper and the chamber walls (inset). **b)** Average generated pressures by the same chips, but reinforced by sealing the filter paper pump tip with transfer tape (inset). The shaded area represents 1 STD (n=4).

S8 Proof-of-concept sampling patch: flow rates

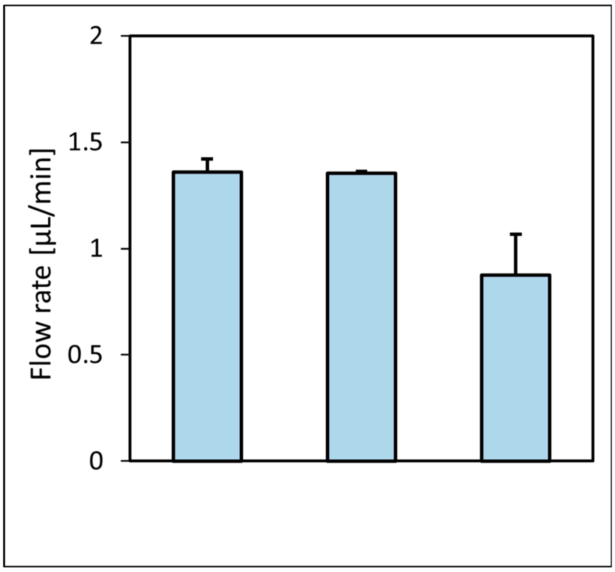

Figure S8: Bar chart depicting the average measured sampling flow rates for water, glycerol-water blood equivalent and sampling from a 2.65% (w/v) agarose gel as human skin equivalent. Error bars represent one standard deviation (n=3).

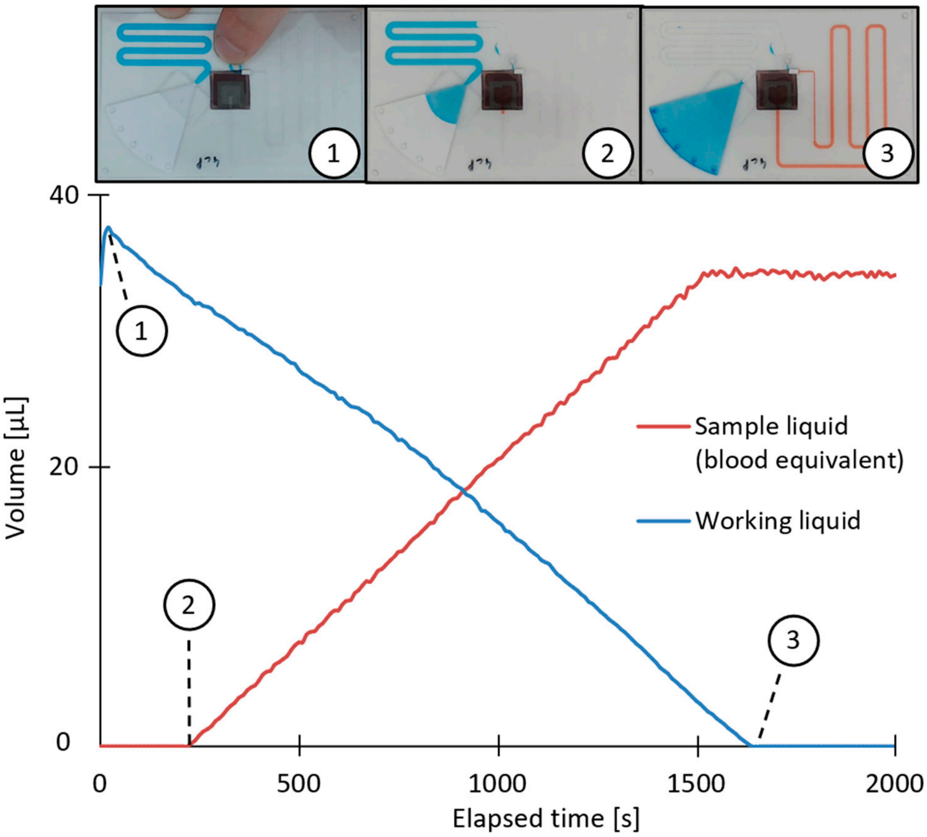

Figure S9: Volume-to-time graph of working liquid (blue) and blood mimicking sample liquid (red). The insets show snapshots for three different moments during operation of the microsampling cartridge: (1) Activation of the SIMPLE unit, (2) Start measuring of the in-channel sample liquid, (3) end of the microsampling operation when all the working liquid has been consumed. The chart shows an almost linearly evolving volume change during microsampling, both for the working liquid and for the sample liquid. It must be remarked that only the sample liquid within the channel was measured as a result of the low resolution of sample liquid on top of the HMNA. The reported total sampled volumes in the manuscript have been corrected based on the designed volume of the chamber on top of the HMNA.
